# Pro-inflammatory dietary patterns and their association with cardiovascular disease and pancreatic cancer in a hospital population: a cross-sectional study

**DOI:** 10.3389/fnut.2025.1666682

**Published:** 2026-01-21

**Authors:** Qiangsong Jin, Yujin Liu, Qiang Tong, Biao Tang, Feng Liang, Lingling Zeng

**Affiliations:** 1 Department of Cardiovascular Medicine, Affiliated Jinhua Hospital Zhejiang University School of Medicine, Jinhua, China; 2 Nursing Department, the Second Hospital of Jinhua, Jinhua, China; 3 Department of Gastroenterology, Huai'an Second People's Hospital, The Affiliated Huai'an Hospital of Xuzhou Medical University, Huai'an, Jiangsu, China; 4 Huai'an Clinical Medical College of Jiangsu University, Huaian Hospital of Huaian City, Huai'an, China

**Keywords:** cardiometabolic risk, cardiovascular disease, pancreatic cancer, patient inflammatory diet score (PIDS), pro-inflammatory dietary pattern, systemic inflammation

## Abstract

**Objective:**

To examine whether a concise, clinic-feasible Patient Inflammatory Diet Score (PIDS) relates to prevalent pancreatic cancer and cardiovascular disease (CVD) in a hospital population, and to explore associations with systemic inflammation.

**Methods:**

We conducted a cross-sectional study among 401 adults (≥40 years) attending cardiology, gastroenterology, or oncology services (2018–2022). A 10–12-min questionnaire captured sociodemographics, lifestyle, and habitual intake of pro- and anti-inflammatory food groups to derive the PIDS (quartiles). Pancreatic cancer and CVD were ascertained from de-identified electronic records; high-sensitivity C-reactive protein (hsCRP) indexed systemic inflammation. Robust Poisson models estimated prevalence ratios (PRs) across PIDS quartiles with prespecified adjustments and subgroup/sensitivity analyses.

**Results:**

Pancreatic cancer was present in 24 participants (6.0%); CVD in 111 (27.7%). Relative to Q1, fully adjusted PRs for pancreatic cancer were 0.81 (95% CI 0.32–2.06), 0.94 (0.39–2.27), and 1.09 (0.45–2.65) for Q2–Q4 (p-trend = 0.79); the per-SD estimate was 1.03 (0.81–1.31). PIDS showed no material association with prevalent CVD (Q4 vs. Q1, PR 1.08; 0.76–1.54; p-trend = 0.61). Correlation with hsCRP was weak (*ρ* = 0.09; *p* = 0.08), and findings were consistent across sex, age, and BMI strata, alternative PIDS categorizations, exclusion of hsCRP > 10 mg·L^−1^, and restriction to participants without CVD. No synergistic effects were observed for joint PIDS–CVD categories.

**Conclusion:**

In this pragmatic clinical setting, a brief, food-based inflammatory diet score did not discriminate cross-sectional differences in pancreatic-cancer prevalence or CVD, nor did it correlate meaningfully with hsCRP. These null findings bound plausible effect sizes and support the need for larger, prospective studies with richer dietary phenotyping and biomarker integration.

## 1 Introduction

Pancreatic cancer remains a highly lethal malignancy, with late presentation and limited opportunities for effective screening or early detection. Identifying pragmatic, clinic-feasible markers that capture modifiable risk processes is therefore a priority (1–4). Chronic, low-grade inflammation has been implicated in pancreatic carcinogenesis through multiple pathways, including cytokine signalling, oxidative stress, insulin resistance, and alterations in the tumour microenvironment (5–7). Diet is a major, modifiable determinant of systemic inflammatory tone, and population studies have reported that patterns characterised by higher intakes of refined grains, processed meats, and sugar-sweetened beverages—and lower intakes of fruits, vegetables, legumes, nuts, and marine fish—are associated with a more pro-inflammatory milieu (8–11). In this manuscript, we use the term “inflammatory diet” to describe an overall pattern of food intake that, based on prior epidemiologic and experimental evidence, is expected to promote higher levels of circulating inflammatory markers and related cardiometabolic disturbances, whereas an “anti-inflammatory” diet reflects the converse pattern. However, translation of these observations to routine clinical settings has been limited by the length and complexity of traditional dietary instruments and by the fact that commonly used research indices of dietary inflammatory potential such as the Dietary Inflammatory Index (DII) and related scores—require detailed nutrient-level data and non-trivial computation that are difficult to embed in usual care. The DII, for example, combines multiple food and nutrient parameters, weighted against literature-derived inflammatory coefficients, to yield a continuous score; this makes it well suited for large epidemiological cohorts but less practical for rapid risk appraisal in busy clinics.

Cardiovascular disease (CVD) and cancer also share inflammatory and metabolic pathways, and CVD commonly coexists with cancer in real-world practice (12–17). This intersection provides an opportunity to examine whether a simple indicator of the inflammatory potential of habitual diet is informative not only for cancer risk stratification but also for cardiometabolic profiling within integrated care pathways (10, 18, 19).

Against this backdrop, we developed a brief, patient-administered questionnaire that yields a food-based Patient Inflammatory Diet Score (PIDS) suitable for use during routine visits, conceptually aligned with DII-type indices but operationalised at the level of a small number of food groups rather than detailed nutrients, and linked these data to de-identified clinical records. This design was chosen to maximise feasibility, reduce respondent burden, and reflect the information typically available to clinicians. Thus, PIDS is intended as a pragmatic, clinic-ready tool that translates the notion of pro- versus anti-inflammatory dietary patterns into a format that can be obtained within a single outpatient consultation, rather than as a substitute for comprehensive research indices.

The primary objective of this study was to evaluate the cross-sectional association between the inflammatory potential of habitual diet, indexed by the PIDS, and the prevalence of pancreatic cancer in a hospital population. Secondary objectives were to assess the relationships of PIDS with prevalent CVD and with a systemic inflammatory biomarker (high-sensitivity C-reactive protein), to explore subgroup consistency, and to examine potential joint effects of PIDS and CVD. We hypothesised that higher PIDS values would be associated with higher prevalence of pancreatic cancer, higher prevalence of CVD, and higher hsCRP levels.

## 2 Method

### 2.1 Study design and participants

We conducted a hospital-based, cross-sectional study that combined a brief patient questionnaire with linkage to de-identified clinical records from the Affiliated Jinhua Hospital Zhejiang University School of Medicine. Between January 2018 and December 2022, consecutive adults attending cardiology, gastroenterology, or oncology clinics/wards were approached. Eligibility criteria were: age ≥ 40 years, ability to complete the questionnaire independently or with staff assistance, and availability of core clinical data in the electronic health record (EHR). We excluded patients with documented cognitive impairment precluding reliable self-report, current pregnancy, or missing key variables (>20% of questionnaire items or absent diagnostic/anthropometric data in the EHR). The final analytic sample comprised 401 patients. The study complied with the Declaration of Helsinki and was approved by the Institutional Review Board of the Huai’an Second People’s Hospital (approval No. 2023043). Written informed consent was obtained for questionnaire participation.

### 2.2 Data sources and measures

#### 2.2.1 Patient questionnaire and dietary score

Trained researchers administered a 10–12-min questionnaire at the index visit. The instrument captured sociodemographics (age, sex, education, marital status), lifestyle factors (current smoking, alcohol use, self-reported weekly moderate-to-vigorous physical activity), and habitual diet using a concise food-frequency module tailored for clinical use. The module queried usual intake over the past 3 months of common pro-inflammatory and anti-inflammatory food groups in Chinese diets (five response options: “rarely/never,” “<1 time/week,” “1–3 times/week,” “4–6 times/week,” “≥1 time/day”). In the study region in eastern China, staple dietary patterns typically include polished rice and wheat-based noodles as primary carbohydrates, frequent consumption of pork and processed meats, and ready access to deep-fried snacks and sugar-sweetened beverages in urban settings, alongside regular intake of vegetables, soy products and pickled dishes and more variable consumption of marine fish depending on proximity to coastal markets.

A simple, food-based Patient Inflammatory Diet Score (PIDS) was pre-specified to index the dietary inflammatory potential without biomarker modelling, drawing conceptually on DII-type constructs while being purposely simplified for routine clinical implementation. Pro-inflammatory items (refined grains/noodles, processed meats, red meats, sugar-sweetened beverages, deep-fried foods) were scored 0–4 points from lowest to highest frequency; anti-inflammatory items (leafy vegetables, other vegetables, fresh fruit, legumes/soy products, nuts, and marine fish/seafood) were reverse-scored 4–0. The PIDS was calculated as the unweighted sum across items (higher values denote greater inflammatory potential) and grouped into quartiles for analysis, an approach chosen to preserve transparency at the point of care, avoid overfitting in this modest dataset, and reflect the absence of robust, population-specific effect estimates that would justify differential weighting. Item selection and scoring directionality were informed by prior literature on the DII and related scores, Chinese dietary guidelines, and local dietary patterns, prioritising food groups that are both empirically linked to systemic inflammation and readily recognisable by patients in brief counselling encounters. Operationally, lower PIDS values correspond to frequent consumption of vegetables, fruit, legumes/soy products, nuts and marine fish with infrequent intake of refined grains, processed and red meats, sugar-sweetened beverages and deep-fried foods, whereas higher values capture the converse pattern. We did not compute the DII itself because nutrient-level intakes were not available from this brief food-frequency module, and our intention was to test whether a much simpler, food-group-based summary retained any discriminatory capacity in a real-world hospital setting.

#### 2.2.2 Ascertainment of pancreatic cancer and cardiovascular disease

Outcomes and comorbidities were obtained from the EHR and then de-identified before analysis. Pancreatic cancer status (primary outcome) was determined by histopathology or, when applicable, by multidisciplinary consensus based on imaging and clinical documentation (ICD-10 C25.x). Cardiovascular disease (CVD) was defined as a documented history of myocardial infarction, coronary revascularisation, heart failure, or ischaemic/haemorrhagic stroke recorded by treating physicians. When both pancreatic cancer and CVD were present, the earliest recorded diagnosis date was retained for descriptive tabulation only.

#### 2.2.3 Clinical measurements and other covariates

From the EHR we extracted height, weight (to compute BMI, kg·m^−2^), resting blood pressure (mean of two automated readings after ≥5 min seated rest), fasting plasma glucose, lipid panel, high-sensitivity C-reactive protein (hsCRP), and serum creatinine. Estimated glomerular filtration rate (eGFR) was derived using the CKD-EPI equation. Medication use (antihypertensives, statins, antidiabetics) and physician-documented hypertension, diabetes and dyslipidaemia were recorded. We considered cardiometabolic conditions such as hypertension, diabetes, dyslipidaemia, reduced kidney function and established CVD as conceptually important comorbidities because they may both influence dietary choices (for example, salt, fat and sugar intake) and reflect underlying systemic inflammation; these variables were therefore used to characterise the sample, and CVD status was incorporated as a covariate in the most fully adjusted models. Data linkage to survey responses used the medical-record number within a secure environment; a unique study ID was then assigned and all direct identifiers were removed prior to analysis.

### 2.3 Statistical analysis

Continuous variables are summarised as mean ± standard deviation (SD) or median (interquartile range, IQR), and categorical variables as number (percentage). Baseline characteristics were compared across PIDS quartiles using one-way ANOVA or Kruskal–Wallis tests for continuous variables and *χ*^2^ tests for categorical variables.

The primary analysis estimated prevalence ratios (PRs) for pancreatic cancer across PIDS quartiles using Poisson regression with robust variance, with linear trend tested by modelling quartiles as an ordinal variable. We also modelled PIDS as a continuous variable (per SD). A parsimonious adjustment strategy was pre-specified:

1. Model 1: age and sex;
2. Model 2: Model 1 + BMI, smoking, alcohol use, physical activity, education;
3. Model 3 (attenuation check): Model 2 + CVD (yes/no).

In secondary analyses, we examined the association of PIDS with prevalent CVD (same modelling framework) and explored correlation between PIDS and hsCRP (Spearman’s rho). Prespecified subgroup analyses evaluated consistency by sex, age (<60 vs. ≥ 60 years), and BMI (<24 vs. ≥ 24 kg·m^−2^) using stratified models; interaction terms tested heterogeneity. Missing data were handled by complete-case analysis, whereby participants with missing values for any exposure, covariate or outcome in a given model were excluded; this approach preserves internal consistency of the multivariable adjustment set but assumes that, conditional on observed data, missingness is independent of pancreatic cancer and CVD status. Two-sided *p* < 0.05 indicated statistical significance. Analyses were performed in R (version 4.3.2).

## 3 Results

### 3.1 Study population and baseline characteristics

From January 2018 to December 2022, 496 patients were approached; 95 were excluded (declined participation, *n* = 54; cognitive impairment, *n* = 13; pregnancy, *n* = 6; missing key data, *n* = 22), yielding 401 participants for analysis. Pancreatic cancer was present in 24 patients (6.0%); established CVD in 111 (27.7%). As shown in Table 1, patients with pancreatic cancer were modestly older (63.7 ± 9.6 y vs. 61.0 ± 10.9 y), more often female (54.2% vs. 44.3%), and had slightly higher hsCRP (median [IQR], 2.4 [1.3–4.0] vs. 1.8 [0.9–3.0] mg·L^−1^) than those without cancer; none of these differences reached statistical significance (all *p* > 0.05). Prevalent CVD was more frequent among cases (37.5%) than non-cases (27.1%), *p* = 0.27.

**Table 1:** Baseline characteristics by pancreatic cancer status (*n* = 401).

Characteristic	Pancreatic cancer (*n* = 24)	No pancreatic cancer (*n* = 377)	*p-*value
Age, years (mean ± SD)	63.7 ± 9.6	61.0 ± 10.9	0.21
Female sex, *n* (%)	13 (54.2)	167 (44.3)	0.31
BMI, kg·m^−2^ (median [IQR])	24.6 [22.7–26.5]	24.3 [22.4–26.1]	0.38
hsCRP, mg·L^−1^ (median [IQR])	2.4 [1.3–4.0]	1.8 [0.9–3.0]	0.2
Current smoker, *n* (%)	8 (33.3)	101 (26.8)	0.49
Current alcohol use, *n* (%)	7 (29.2)	108 (28.6)	0.94
≥150 min·wk^−1^ physical activity, *n* (%)	10 (41.7)	171 (45.4)	0.72
Education ≥ high school, *n* (%)	16 (66.7)	259 (68.7)	0.83
Prevalent CVD, *n* (%)	9 (37.5)	102 (27.1)	0.27

The Patient Inflammatory Diet Score (PIDS) had a median of 20 (IQR 15–26); quartile cut-points were 13, 19 and 26. In this scoring scheme, values in the lowest quartile typically reflect frequent intake of several anti-inflammatory food groups (e.g., vegetables, fruit, legumes/soy products and marine fish) with only occasional consumption of pro-inflammatory items, whereas values in the highest quartile indicate more frequent intake of refined grains, processed and red meats, sugar-sweetened beverages and deep-fried foods together with comparatively lower intake of protective foods. Across ascending quartiles (Q1–Q4: *n* = 100, 100, 100, 101), baseline characteristics were broadly similar (Table 2). Mean age (60.8–62.0 y) and BMI (median 23.9–24.6 kg·m^−2^) showed only small, non-significant increases (p-trend = 0.67 and 0.09, respectively). The proportion of women declined from 50.0% in Q1 to 37.6% in Q4 (p-trend = 0.12). Median hsCRP rose slightly from 1.7 to 2.0 mg·L^−1^ (p-trend = 0.08). The crude prevalence of CVD was relatively flat (26.0, 27.0, 28.0, and 29.7% across Q1–Q4, p-trend = 0.55). Pancreatic-cancer prevalence varied minimally by PIDS quartile—6.0, 5.0, 6.0, and 6.9%—without evidence of a gradient (p-trend = 0.77).

**Table 2:** Baseline characteristics across PIDS quartiles (Q1 ≤ 13; Q2 = 14–19; Q3 = 20–26; Q4 ≥ 27 points).

Characteristic	Q1 (*n* = 100)	Q2 (*n* = 100)	Q3 (*n* = 100)	Q4 (*n* = 101)	*p* for trend
Age, years (mean ± SD)	60.8 ± 10.5	61.2 ± 10.7	61.6 ± 10.9	62.0 ± 10.8	0.67
Female sex, *n* (%)	50 (50.0)	46 (46.0)	46 (46.0)	38 (37.6)	0.12
BMI, kg·m^−2^ (median [IQR])	23.9 [22.3–25.9]	24.1 [22.5–26.1]	24.4 [22.6–26.4]	24.6 [22.7–26.5]	0.09
hsCRP, mg·L^−1^ (median [IQR])	1.7 [0.9–2.9]	1.8 [0.9–3.0]	1.9 [1.0–3.1]	2.0 [1.1–3.2]	0.08
Prevalent CVD, *n* (%)	26 (26.0)	27 (27.0)	28 (28.0)	30 (29.7)	0.55
Pancreatic cancer, *n* (%)	6 (6.0)	5 (5.0)	6 (6.0)	7 (6.9)	0.77

### 3.2 Primary analysis: PIDS and pancreatic cancer

**Figure 1:**
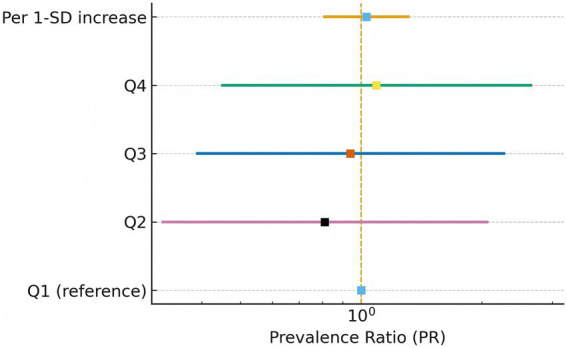
Adjusted prevalence ratios for pancreatic cancer by PIDS quartile and per-SD increase.

Multivariable Poisson models did not identify a statistically significant association between PIDS and pancreatic cancer (Table 3). Using Q1 as reference, fully adjusted prevalence ratios (PRs; Model 3) were 0.81 (95% CI 0.32–2.06) for Q2, 0.94 (0.39–2.27) for Q3, and 1.09 (0.45–2.65) for Q4 (p-trend = 0.79). Modelling PIDS as a continuous variable yielded a PR per 1-SD increase of 1.03 (0.81–1.31). Results were similar in age- and sex-adjusted (Model 1) and lifestyle-adjusted (Model 2) specifications, with all confidence intervals crossing unity. The corresponding forest plot of adjusted estimates is shown in Figure 1.

**Table 3:** Association between PIDS and pancreatic cancer (robust Poisson models).

PIDS category	Cases/total (%)	Model 1 PR (95% CI)	Model 2 PR (95% CI)	Model 3 PR (95% CI)
Q1	6/100 (6.0)	1	1	1
Q2	5/100 (5.0)	0.83 (0.33–2.12)	0.82 (0.32–2.09)	0.81 (0.32–2.06)
Q3	6/100 (6.0)	0.98 (0.41–2.33)	0.96 (0.40–2.30)	0.94 (0.39–2.27)
Q4	7/101 (6.9)	1.12 (0.47–2.73)	1.10 (0.46–2.68)	1.09 (0.45–2.65)
*p* for trend	–	0.78	0.78	0.79
Per 1-SD higher PIDS	–	1.04 (0.83–1.30)	1.03 (0.82–1.30)	1.03 (0.81–1.31)

Model 1 = age, sex; Model 2 = Model 1 + BMI, smoking, alcohol, physical activity, education; Model 3 = Model 2 + CVD (yes/no).

### 3.3 Secondary analyses: CVD and inflammatory biomarkers

In parallel models for prevalent CVD (Table 4), PIDS quartiles were not materially associated with CVD after adjustment (Q4 vs. Q1, PR 1.08; 95% CI 0.76–1.54; p-trend = 0.61). Correlation analyses between PIDS and hsCRP indicated only weak relationships (Table 5). Spearman’s *ρ* was 0.09 (*p* = 0.08) overall. When hsCRP was dichotomised at 3 mg·L^−1^, the fully adjusted PR for Q4 vs. Q1 was 1.18 (0.74–1.88, p-trend = 0.39), consistent with the small mean/median differences seen across quartiles.

**Table 4:** Association between PIDS and prevalent cardiovascular disease (robust Poisson models).

PIDS category	CVD cases/total (%)	Model 1 PR (95% CI)	Fully adjusted PR (95% CI)
Q1 (reference)	26/100 (26.0)	1	1
Q2	27/100 (27.0)	1.01 (0.75–1.36)	1.02 (0.76–1.38)
Q3	28/100 (28.0)	1.04 (0.77–1.39)	1.04 (0.78–1.40)
Q4	30/101 (29.7)	1.07 (0.77–1.50)	1.08 (0.76–1.54)
*p* for trend	–	0.62	0.61
Per 1-SD higher PIDS	–	1.03 (0.94–1.14)	1.04 (0.94–1.15)

Model 1 = age, sex; fully adjusted = Model 1 + BMI, smoking, alcohol, physical activity, education.

**Table 5:** Relationship between PIDS and systemic inflammation (hsCRP).

PIDS category	hsCRP ≥3 mg·L^−1^/total (%)	Fully adjusted PR (95% CI)
Q1	21/100 (21.0)	1
Q2	24/100 (24.0)	1.06 (0.71–1.60)
Q3	26/100 (26.0)	1.12 (0.75–1.68)
Q4	30/101 (29.7)	1.18 (0.74–1.88)
*p* for trend	–	0.39

Fully adjusted = age, sex, BMI, smoking, alcohol, physical activity, education.

### 3.4 Sensitivity analyses

Findings were robust to several checks (Table 6). Results were unchanged when PIDS was analysed in tertiles (Q3 vs. Q1, PR 1.05; 95% CI 0.50–2.23), when participants with hsCRP > 10 mg·L^−1^ (*n* = 19) were excluded (Q4 vs. Q1, PR 1.03; 0.37–2.83; p-trend = 0.88), and when restricting the sample to those without prevalent CVD (*n* = 290; Q4 vs. Q1, PR 1.05; 0.33–3.36; p-trend = 0.84).

**Table 6:** Sensitivity analyses for PIDS and pancreatic cancer.

Analysis specification	Sample (*n*)	Cases (*n*)	Comparison	Adjusted PR (95% CI)	*p* for trend
PIDS in tertiles	401	24	T3 vs. T1	1.05 (0.50–2.23)	0.83
Excluding hsCRP >10 mg·L^−1^	382	23	Q4 vs. Q1	1.03 (0.37–2.83)	0.88
Restricting to participants without CVD	290	15	Q4 vs. Q1	1.05 (0.33–3.36)	0.84

All models adjusted as in Model 2.

### 3.5 Subgroup analyses

Prespecified subgroup analyses (Table 7) showed no evidence of meaningful effect modification. Fully adjusted PRs (Q4 vs. Q1) were 1.08 (0.31–3.74) in men and 1.09 (0.32–3.70) in women (p-interaction = 0.99). Estimates were similarly null for age < 60 vs. ≥ 60 years (0.98 [0.28–3.45] vs. 1.18 [0.42–3.35]; p-interaction = 0.78) and BMI < 24 vs. ≥ 24 kg·m^−2^ (1.03 [0.31–3.44] vs. 1.12 [0.40–3.13]; p-interaction = 0.88).

**Table 7:** Subgroup analyses for PIDS (Q4 vs. Q1) and pancreatic cancer.

Subgroup	Adjusted PR (95% CI)	*p* for interaction
Sex		
Men	1.08 (0.31–3.74)	0.99
Women	1.09 (0.32–3.70)	
Age		
<60 years	0.98 (0.28–3.45)	0.78
≥60 years	1.18 (0.42–3.35)	
BMI		
<24 kg·m^−2^	1.03 (0.31–3.44)	0.88
≥24 kg·m^−2^	1.12 (0.40–3.13)	

Model adjusted as in Model 2.

### 3.6 Joint categorisation of PIDS and CVD

A cross-classification of PIDS (low: Q1–Q2; high: Q3–Q4) and CVD (no/yes) did not reveal synergistic effects on pancreatic-cancer prevalence (Table 8). Relative to low-PIDS/no-CVD (reference), adjusted PRs were 1.21 (0.52–2.82) for low-PIDS/with-CVD, 1.07 (0.44–2.60) for high-PIDS/no-CVD, and 1.43 (0.60–3.39) for high-PIDS/with-CVD (p-interaction = 0.74).

**Table 8:** Joint categorisation of PIDS and CVD in relation to pancreatic cancer.

Group (reference = low PIDS and No CVD)	Total *n*	Pancreatic cancer, *n* (%)	Adjusted PR (95% CI)
Low PIDS (Q1–Q2) and No CVD	147	7 (4.8)	1
Low PIDS (Q1–Q2) and CVD	53	4 (7.5)	1.21 (0.52–2.82)
High PIDS (Q3–Q4) and No CVD	143	8 (5.6)	1.07 (0.44–2.60)
High PIDS (Q3–Q4) and CVD	58	5 (8.6)	1.43 (0.60–3.39)
*p* for PIDS × CVD interaction	–	–	0.74

The questionnaire-based dietary score showed only weak, non-significant relations with both pancreatic cancer and CVD in this sample of 401 patients, and associations did not strengthen under alternative specifications or within subgroups.

## 4 Discussion

In this hospital-based, cross-sectional study that paired a brief patient questionnaire with de-identified clinical records, we found no statistically significant association between the Patient Inflammatory Diet Score (PIDS) and pancreatic cancer. Across PIDS quartiles, adjusted prevalence ratios (PRs) hovered around unity (Q4 vs. Q1, PR 1.09; 95% CI 0.45–2.65), and the per-SD estimate was similarly null (PR 1.03; 0.81–1.31). In absolute terms, pancreatic-cancer prevalence differed little between the extreme quartiles (6.0% in Q1 vs. 6.9% in Q4), indicating that even individuals with comparatively pro-inflammatory dietary patterns, as captured by PIDS, did not have markedly higher case prevalence in this sample. Results were concordantly negative for prevalent cardiovascular disease (CVD) (Q4 vs. Q1, PR 1.08; 0.76–1.54) and for correlations with hsCRP, and they did not vary meaningfully in prespecified subgroups or sensitivity analyses. These findings suggest that—in the present clinical setting and with a pragmatic, patient-administered instrument that intentionally sacrifices some granularity relative to indices such as the DII—the inflammatory potential of habitual diet offers limited discriminative value for cross-sectional differences in pancreatic-cancer prevalence.

Our results contrast with prior reports that linked pro-inflammatory dietary patterns with higher pancreatic-cancer risk in cohort-based settings. Multiple studies, primarily from Western populations, have implicated refined grains, processed meats, and sugar-sweetened beverages, alongside low intakes of fruits, vegetables and marine *n*-3 fatty acids, in promoting a systemic inflammatory milieu conducive to pancreatic carcinogenesis (20–25). Evidence from other cohorts has buttressed this connection and suggested cultural generalisability, including studies set in non-Chinese populations and large administrative databases (26–28). Most of these studies have operationalised dietary inflammatory potential using nutrient-based indices such as the DII, whereas our PIDS applies a food-group-based, equally weighted scoring system aimed at maximising feasibility in clinical encounters rather than replicating the full complexity of research instruments. Several factors may explain the discrepancy. Design differences—our cross-sectional approach versus longitudinal follow-up—limit temporal inference and reduce sensitivity to small effects that accrue over time (29–31). Exposure measurement in the current study relied on a concise food-frequency module that purposely traded depth for feasibility, likely increasing non-differential misclassification and biasing associations toward the null. Range restriction in dietary contrast within a single hospital catchment may have narrowed between-person variability compared with community cohorts (32–35). Our modest case count (*n* = 24) constrained precision around stratified estimates. These explanations are consistent with the broader literature and underscore the need for larger, prospective designs when interrogating diet–cancer relations.

The absence of a clear gradient for CVD or evidence of PIDS × CVD interaction (Table 8) should also be interpreted in context. Mechanistic and epidemiologic work has highlighted bidirectional ties between vascular pathology and malignancy, with shared inflammatory and metabolic pathways (36–39) and excess cancer incidence reported among survivors of myocardial infarction or heart failure (40–42). Plausible biological links—hyperinsulinaemia/IGF-1 signalling, oxidative stress, and cytokine activation—remain intact even when cross-sectional associations are weak (43–45). Endothelial dysfunction and microvascular hypoxia may shape the pancreatic micro-environment, while microbiome-derived metabolites and bile-acid signalling can influence tumour-immune surveillance (46–48). In our setting, imprecision in brief dietary assessment, survivor and referral biases inherent to a hospital sample, and limited statistical power together may have masked small-to-moderate joint effects that would require larger samples or longitudinal follow-up to detect.

Despite negative findings, several practical lessons emerge. A short, clinic-feasible dietary screen such as the PIDS may not suffice for risk stratification of pancreatic cancer at the point of care; however, it can still support general cardiometabolic counselling while more definitive evidence accumulates. For example, in routine cardiology or oncology visits, PIDS could be completed by patients while waiting, automatically scored within the electronic health record, and used to flag those with particularly pro-inflammatory eating patterns for brief, structured dietary counselling or referral to dietetic services. Repeated administration over time could also provide a simple, low-burden means of monitoring diet-related behaviour change alongside blood pressure, lipids and glycaemic indices within integrated cardio-oncology or chronic-disease management pathways. Integrative cardio-oncology models—coordinating cardiovascular prevention with vigilant cancer surveillance—remain sensible given shared mechanisms, even if diet-based screening alone is not discriminatory in this context (49, 50). The null results help bound plausible effect sizes for future studies and trial planning: detectable diet–cancer associations in similar hospital populations are likely modest and will require larger samples, richer exposure ascertainment (e.g., repeated measures, device-assisted recalls), and objective biomarkers of inflammation and metabolism to minimise misclassification. Given ongoing work on inflammatory and endothelial pathways in both CVD and pancreatic cancer, lifestyle modification aimed at reducing systemic inflammation remains a low-risk, high-yield priority for cardiovascular health, even if its direct cross-sectional signal for pancreatic cancer is small in the present data.

This study has important limitations that contextualise the null results. (1) Cross-sectional design: temporality cannot be established, and reverse causation is a particular concern for pancreatic cancer, because pre-diagnostic symptoms and treatment can prompt substantial diet changes, so disease-related dietary changes cannot be excluded even though diagnosis dates were aligned for descriptive tabulation only. For example, upper abdominal pain, early satiety, steatorrhoea, unintended weight loss and treatment-related nausea may lead patients to reduce portion sizes, avoid high-fat or fibrous foods, or preferentially consume refined, easily digested items, so the recorded diet may partly reflect consequences of established disease rather than long-term antecedent patterns. (2) Exposure assessment: the 10–12-min questionnaire captured habitual diet over 3 months and omitted details (portion sizes, cooking methods) available in comprehensive FFQs; non-differential error likely biased PRs toward 1.00. (3) Statistical power: only 24 pancreatic-cancer cases limited precision, especially in subgroups and interaction tests; true small effects may therefore remain undetected. (4) Outcome and comorbidity ascertainment: although obtained from the EHR, misclassification is possible (e.g., incomplete documentation of remote CVD), and hsCRP was measured once. (5) Confounding: residual confounding by unmeasured or incompletely characterised pancreatic-cancer risk factors—such as chronic pancreatitis, detailed smoking intensity and duration, family history of pancreatic cancer, new-onset diabetes and specific occupational exposures (e.g., to pesticides, petroleum products or heavy metals)—cannot be excluded despite parsimonious adjustment. (6) Missing data and analytic choices: complete-case analysis and the upfront exclusion of patients with >20% missing questionnaire items may introduce selection bias if individuals with incomplete dietary data differ systematically in both diet and pancreatic-cancer risk (for example, being more acutely unwell, having lower health literacy or experiencing more severe gastrointestinal symptoms that affect eating patterns), which could attenuate or distort observed associations. (7) Generalisability: single-centre recruitment in Zhejiang limits external validity to other regions and care settings. Using a streamlined, patient-administered dietary instrument linked with clinical records, we observed no material association between inflammatory dietary potential and pancreatic cancer or prevalent CVD. While this negative control does not support PIDS as a stand-alone marker for pancreatic-cancer risk in routine practice, it clarifies design needs for future work and reinforces the prudence of integrated cardiovascular prevention and general nutrition counselling pending larger, prospective evaluations.

## 5 Conclusion

In this hospital-based, cross-sectional study of 401 patients, a brief, patient-administered dietary instrument linked to de-identified clinical data showed no material association between the inflammatory potential of habitual diet and pancreatic cancer. Findings were similarly null for prevalent cardiovascular disease and for systemic inflammation, and they remained stable across sensitivity and subgroup analyses without evidence of interaction between dietary score and cardiovascular comorbidity. Taken together, these results indicate that a concise dietary screen has limited discriminatory value for pancreatic-cancer risk stratification at the point of care. For clinical practice, the data do not support diet-based screening to identify individuals with pancreatic cancer. Nonetheless, routine dietary assessment remains useful for cardiometabolic counselling, where benefits are well established, and can be embedded within coordinated cardio-oncology pathways. Future work should prioritise adequately powered, preferably multicentre prospective designs; richer dietary phenotyping with repeated measures; and incorporation of objective biomarkers of inflammation and metabolism to reduce non-differential misclassification and strengthen causal inference. Attention to residual confounding, range restriction within clinical settings, and transparent, pre-specified analyses will be essential. Evaluation of composite risk models that integrate lifestyle, metabolic, and clinical predictors may prove more informative than diet alone. Specifically, future research should include: (1) large, multicentre prospective cohort studies with adjudicated pancreatic-cancer outcomes and repeated, validated assessments of dietary inflammatory potential; (2) nested case–control or case–cohort studies that incorporate panels of inflammatory, metabolic and microbiome-related biomarkers to interrogate mechanisms; and (3) intervention trials that target pro-inflammatory dietary patterns and test their impact on systemic inflammatory markers and intermediate pancreatic and cardiovascular end points. While the present results are largely negative, they provide clear boundaries for plausible effect sizes and practical guidance for study design, supporting a shift toward larger longitudinal cohorts that combine detailed exposure assessment with mechanistic biomarkers to clarify any role of inflammatory dietary patterns in pancreatic carcinogenesis.

## Data Availability

The original contributions presented in the study are included in the article/supplementary material, further inquiries can be directed to the corresponding authors.
